# Supplementary material for: A Robust DNA Isolation Protocol from Filtered Commercial Olive Oil for PCR-Based Fingerprinting
Source: Foods. 2019 Oct 9;8(10):462. doi: 10.3390/foods8100462 (PMC6836273; doi:10.3390/foods8100462)
Supplement: Supplementary file 1 [file foods-08-00462-s001.zip › TableS1.docx]

| **Locus** | **Repeated motif** | **Primer sequence (5'🡺3')** | **Annealing temperature (°C)** |
| --- | --- | --- | --- |
| **DCA03** | (GA)_19_ | cccaagcggaggtgtatattgttac | 50 |
|  |  | tgcttttgtgtttgagatgttg |  |
| **DCA18** | (CA)_4_CT(CA)_3_(GA)_19_ | aagaaagaaaaaggcagaattaagc | 50 |
|  |  | gttttcgtctctctacataagtgac |  |
|  |  |  |  |
| **DCA04** | (GA)_16_ | cttaactttgtgcttctccatatcc | 55 |
|  |  | agtgacaaaagcaaaagactaaagc |  |
|  |  |  |  |
| **DCA09** | (GA)_23_ | aatcaaagtcttccttctcatttcg | 55 |
|  |  | gatccttccaaaagtataacctctc |  |
|  |  |  |  |
| **DCA05** | (GA)_15_ | aacaaatcccatacgaactgcc | 50 |
|  |  | cgtgttgctgtgaagaaaatcg |  |
|  |  |  |  |
| **DCA15** | (CA)_3_G(AC)_14_ | gatcttgtctgtatatccacac | 50 |
|  |  | tataccttttccatcttgacgc |  |
|  |  |  |  |
| **GAPU101** | (GA)_8_(G)_3_(AG)_3_ | catgaaaggagggggacata | 60 |
|  |  | ggcacttgttgtgcagattg |  |
| **EMO90** | (CA)_10_ | catccggatttcttgctttt | 50 |
|  |  | agcgaatgtagctttgcatgt |  |
| **GAPU71b** | GA(AG)_6_(AAG)_8_ | gatcaaaggaagaaggggataaa | 60 |
|  |  | acaacaaatccgtacgcttg |  |
|  |  |  |  |

**Table S1.** List of SSR markers used for EVOO traceability. Primer sequences and annealing temperature are also provided.
