# Supplementary figures and images for: A Robust DNA Isolation Protocol from Filtered Commercial Olive Oil for PCR-Based Fingerprinting
Source: Foods. 2019 Oct 9;8(10):462. doi: 10.3390/foods8100462 (PMC6836273; doi:10.3390/foods8100462)

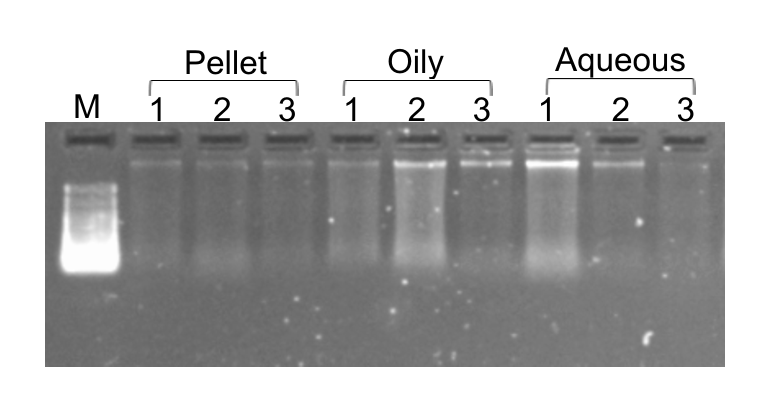

Supplement: Supplementary file 1 [file foods-08-00462-s001.zip › FigureS1.tif]

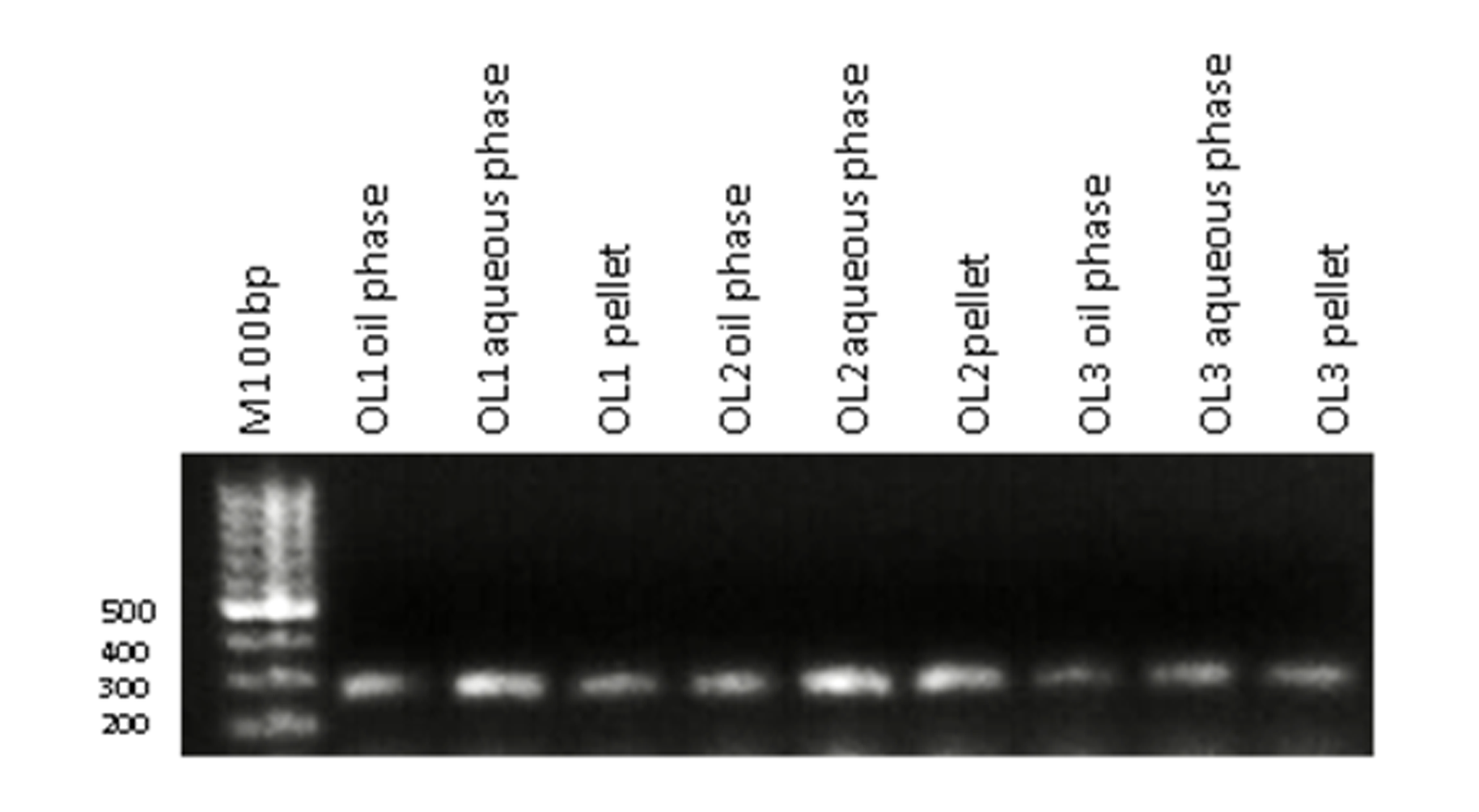

Supplement: Supplementary file 1 [file foods-08-00462-s001.zip › FigureS2.tif]

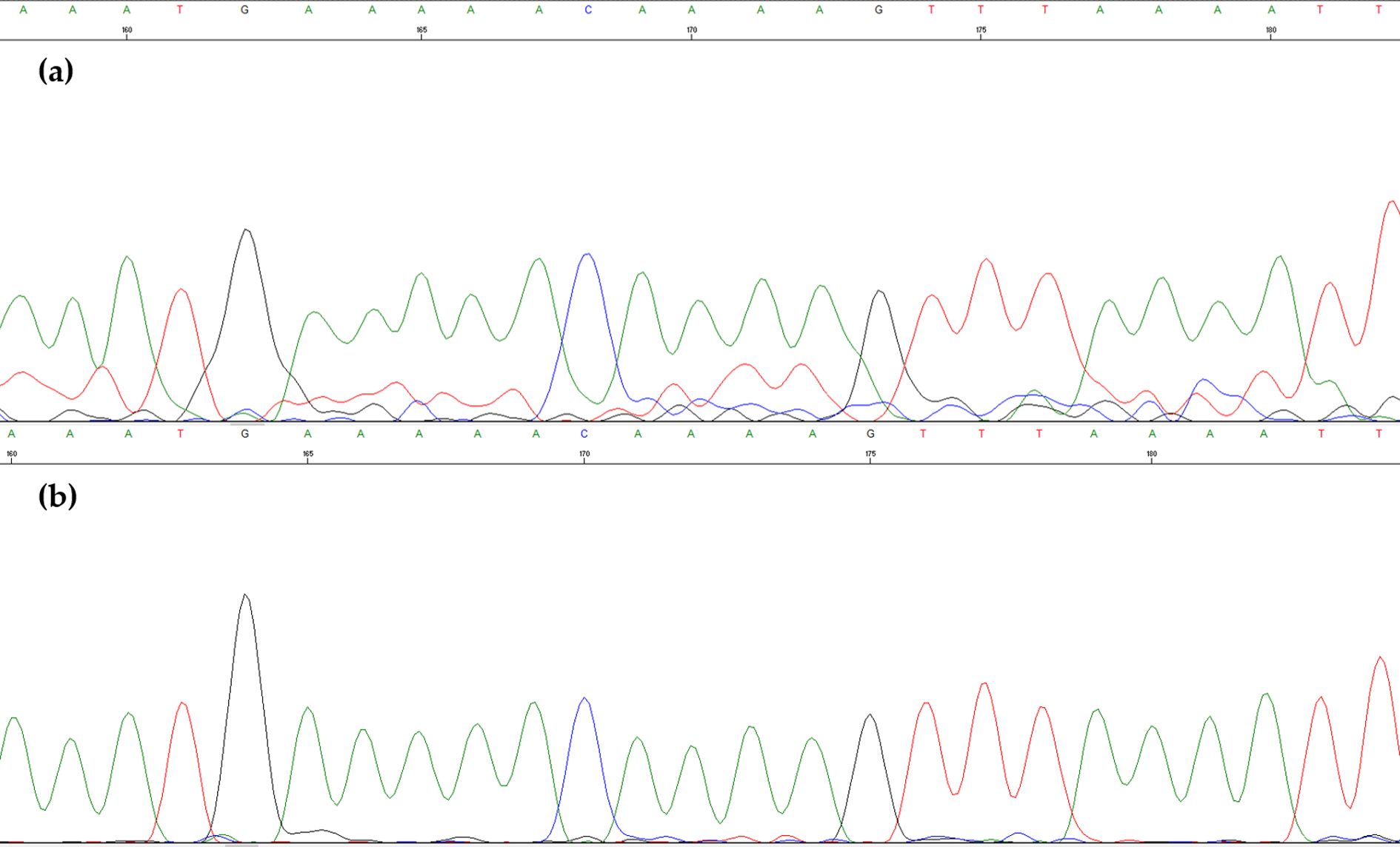

Supplement: Supplementary file 1 [file foods-08-00462-s001.zip › FigureS3.tif]
